# Supplementary material for: Chronic Dehydration in Nursing Home Residents
Source: Nutrients. 2020 Nov 20;12(11):3562. doi: 10.3390/nu12113562 (PMC7709028; doi:10.3390/nu12113562)
Supplement: Supplementary file 1 [file nutrients-12-03562-s001.pdf]

Table S1. Correlation coefficient between eGFR and BMI (Pearson's correlation test).

	**eGFR**	**p-value**
BMI	−0.349	<0.01

eGFR, estimated glomerular filtration rate; BMI, body mass index.
